# Mediating Effect of Perceived Professional Benefit on the Relationship Between Spiritual Health and Spiritual Care Competence Among New Nurses: A Cross-Sectional Study

**DOI:** 10.1155/jonm/8832454

**Published:** 2025-05-02

**Authors:** Cancan Chen, Xiaofei Sun, Yanting Zhang, Zhenya Liu, Miaorui Jiao, Yanli Hu

**Affiliations:** ^1^Henan Provincial Key Medicine Laboratory of Nursing, Henan Provincial People's Hospital, Zhengzhou University People's Hospital, Zhengzhou, Henan, China; ^2^School of Humanities and Design, Zhengzhou Vocational University of Information and Technology, Zhengzhou, Henan, China; ^3^School of Nursing, Zhengzhou Railway Vocational and Technical College, Zhengzhou, Henan, China; ^4^Department of Traditional Chinese Medicine, Henan Cancer Hospital, Zhengzhou, Henan, China; ^5^School of Nursing, Guangzhou Medical University, Guangzhou, China

**Keywords:** nurses, perceived professional benefit, spiritual care competence, spiritual health

## Abstract

**Objectives:** This study aimed to examine the relationship between spiritual health and spiritual care competence among new nurses and explore the mediating role of perceived professional benefit in this relationship.

**Background:** Spiritual care is an integral part of holistic nursing. The ability to deliver spiritual care to patients, known as spiritual care competence, is increasingly being recognized as a crucial occupational skill for nurses, particularly new nurses. Thus, understanding the level of spiritual care competence among new nurses and identifying the factors associated with it have become matters of priority.

**Methods:** In the cross-sectional online study, 299 new nurses were selected using convenience sampling from 10 tertiary hospitals in prefecture-level cities, in Henan Province, China, from March to April 2021. Participants' sociodemographic characteristics, spiritual health, perceived professional benefit, and spiritual care competence were assessed. The mediation model was examined using Model 4 of the PROCESS macro for SPSS.

**Results:** The results showed a positive correlation between spiritual health, perceived professional benefit, and spiritual care competence (both *p* < 0.01). Furthermore, spiritual health had a direct effect on spiritual care competence (effect = 0.187). The association between spiritual health and spiritual care competence was mediated by perceived professional benefit (effect = 0.382).

**Conclusion:** Perceived professional benefit was a mediator in the link between spiritual health and spiritual care competence among new nurses.

**Implications for Nursing Management:** This study's findings underscore the need to promote the spiritual care competence of new nurses in China. Healthcare managers can not only directly promote the spiritual care competence of new nurses by cultivating their spiritual health but also indirectly by enhancing their professional benefits.

## 1. Background

Spiritual health was identified by the World Health Organization as the fourth component of health in 1998, emphasizing the importance of achieving harmony and unity among physical, psychological, social, and spiritual health [1]. In recent years, spirituality has gained considerable attention as an essential and fundamental aspect of health [2–8]. Spirituality refers to the dynamic aspect of human existence that pertains to how people (individually and communally) feel, express, seek meaning, purpose, transcendence, and connect [9]. It plays a crucial role in how individuals cope with life-threatening situations such as illness [10]. Spiritual care involves recognizing and responding to a patient's spiritual needs such as meaningfulness, capacity for self-expression, and faith-based support [11]. Within a team of healthcare professionals, nurses are most likely to encounter patients with spiritual crises and play a vital role in assisting them in confronting their fear of death, decreasing the uncertainty and discomfort of treatment, and restoring their inner peace [12]. Spiritual care is considered the foundation of holistic nursing and has already been integrated into nursing practice [13].

New nurses are those who have recently entered clinical nursing within 2 years of graduation from school [14, 15]. They form a significant part of the healthcare workforce in hospitals. However, new nurses face numerous challenges while navigating the transition to practice [16]. In addition, the lack of spiritual care education and training, coupled with passive attitudes toward spirituality and death during schooling, further complicates the provision of spiritual care by new nurses [17]. Spiritual care competence (SCC) refers to nurses' knowledge, attitudes, and abilities to deliver spiritual practices, including intrapersonal spirituality, interpersonal spirituality, spiritual care assessment and planning, and spiritual care intervention and evaluation [12]. Competence in providing such care is considered a crucial occupational skill for nurses, as it can enhance patients' coping skills, sense of meaning, and overall quality of life [18, 19]. However, in clinical settings, nurses often feel ill-equipped to provide spiritual care because of their limited knowledge and competence [13, 17]. This is especially true for new nurses [20]. Consequently, patients' spiritual needs frequently go unmet [21–23]. Hence, it is crucial to promote the SCC of new nurses, and a potential first step would be to identify modifiable factors and their underlying mechanisms.

Spiritual health is a multidimensional concept, with no consensus on the definition, which is indeed culture-influenced [24]. The term “spirituality” derives from the root “spirit” and corresponds to the Latin spiritus, meaning being alive or life is supreme [25]. Chinese culture, less influenced by Christianity compared to Western countries, is deeply imbued with the philosophies of Confucianism, Buddhism, and/or Daoism [26]. Within Chinese culture, the concept of spirit means “chi,” representing the energy that fulfills the heaven, earth, universe, and nature [27]. Spirituality in China emphasizes achieving inner peace through harmonious connections with oneself, others, and the natural world or higher principles (heavenly principles) [12]. Furthermore, spiritual health, also known as “spiritual well-being,” describes the perception of life's meaning; recognition of one's own value and that of others; harmonious connections with oneself, others, and the environment; possession of internal resources and power; and the ability to adapt to challenging circumstances [28–30]. Heidari's study of 172 Iranian nurses revealed a positive link between spiritual health and their ability to deliver spiritual care, with spiritual health being a predictor of SCC [31]. Wang et al. [32] found that Chinese nurses' SCC was affected by their spiritual health. Spiritual health was required for improved performance in spiritual care practice and served as a protective factor for SCC [33]. However, there is a limited understanding of the relationship between spiritual health and SCC among new nurses, as well as the mechanism of their relationship.

Nurses' perceived professional benefits, in the context of positive psychology, mean the perceived job advantages of the nursing profession and the conviction that nursing can advance people's well-being [34]. The broaden-and-build theory proposes that positive emotional experiences broaden individuals' knowledge and action capability, build personal resources such as physical, spiritual, and social resources, and promote personal growth and development [35]. New nurses encounter increased stress levels during their transition from student-nurse to professional nurse status. This transition entails a significant shift in roles and work settings, leading to feelings of uncertainty, doubt, and lack of clear direction [16, 36]. However, new nurses who effectively use their personal resources to cope with job-related stress gave a positive evaluation of the benefits of their profession [36, 37]. Spiritual health, as a form of nurses' spiritual resource, was not only related to spiritual care but also correlated with positive professional perceptions [38, 39]. In addition, Li et al. [40] reported that perceived professional benefit was positively related to SCC among nurses. Therefore, we hypothesized that perceived professional benefit would mediate the relationship between spiritual health and SCC among new nurses.

**Figure 1:**
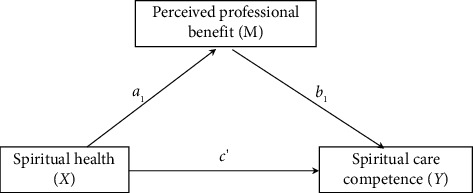
Proposed mediation model of perceived professional benefit in the relationship between spiritual health and spiritual care competence.

According to the broaden-and-build theory, we operationalized spiritual health as an individual resource, perceived professional benefit as a positive emotional experience, and SCC as an action capability to examine the mediating role of perceived professional benefit in the relationship between spiritual health and SCC among new nurses. The aims of our study are as follows: (1) to investigate the levels of SCC in new nurses; (2) to detect the correlations among SCC, spiritual health, and perceived professional benefit; and (3) to test the mediating role of perceived professional benefit between spiritual health and SCC (Figure 1). The findings of this study will inform efforts by healthcare managers to adopt effective interventions to promote SCC among new nurses.

## 2. Methods

### 2.1. Study Design and Participants

In this cross-sectional study, 299 new nurses were recruited using convenience sampling from 10 tertiary hospitals in prefecture-level cities in Henan Province, China. Data were collected from March to April 2021. The inclusion criteria for participants were as follows: (1) acquiring a nursing practice certificate, (2) having worked less than 2 years, (3) direct involvement in providing patient care, and (4) volunteering to participate in this study. Participants who took breaks of 30 days or longer during the survey period were excluded. This study was conducted in accordance with the Declaration of Helsinki and approved by the Ethics Committee of Henan Cancer Hospital (2019014). The study used the Strengthening the Reporting of Observational Studies in Epidemiology (STROBE) guidelines [41] for cross-sectional studies (File S1).

The sample size was computed using G^∗^Power 3.1 software for multiple linear regression considering a moderate effect size of 0.15, a significance level (α) of 0.05, a power of 0.90, and 12 predictor variables [42]. We assumed an invalid questionnaire rate of 20% because of incomplete surveys and short filling time. A sample size of 189 was required. Therefore, 299 participants were considered adequate for this study.

### 2.2. Data Collection

After permission was obtained from the survey hospital administrators, an investigator was assigned to each hospital to facilitate data collection. Notices containing information about the research purpose, estimated survey completion time (approximately 20 min), and inclusion and exclusion criteria were disseminated through instant messaging apps such as WeChat. The informed consent form and instructions for completion appeared on the first page of the electronic questionnaire. Participants were deemed to have given their consent if they completed the questionnaire. The online self-assessment questionnaire was created on the “SO JUMP” platform and could only be submitted successfully if all options were completed. Two researchers carefully reviewed each submitted questionnaire to ensure compliance with the requirements and excluded those that did not meet the criteria.

### 2.3. Measurements

The online self-assessment questionnaire consisted of several sections including nurses' sociodemographic characteristics, the Spiritual Health Scale Short Form, the Perceived Professional Benefit Questionnaire, and the Chinese Version of the Spiritual Care Competence Scale (C-SCCS). Sociodemographic characteristics included sex, age, educational background, marital status, parental status, employment type, monthly income, religious beliefs, and department, which were selected based on the conclusions drawn from previous studies [8, 20, 32, 43–45]. These factors were considered as potential influences on new nurses' SCC and used as control variables in subsequent analyses.

The SCCS developed by van Leeuwen et al. [46] was used to assess nurses' SCC. The C-SCCS was translated by Hu et al. [47] in 2019. The scale contained 27 items covering three dimensions: “assessment, implementation, professionalization and quality improvement of spiritual care”; “personal and team support”; and “attitude toward spirituality and communication of patients.” Participants rated each item on a five-point Likert scale ranging from 1 (strongly disagree) to 5 (strongly agree). Total scores ranged from 27 to 135, with a higher score indicating a higher level of SCC. In this study, Cronbach's alpha for the C-SCCS was 0.982.

The Chinese version of the Spiritual Health Scale Short Form developed by Hsiao et al. [48] was used to assess the nurses' spiritual health. The scale contained 24 items across five aspects: connections to others, meaning derived from living, transcendence, religious attachment, and self-understanding. The scale was rated on a five-point Likert scale ranging from 1 (*strongly disagree*) to 5 (*strongly agree*). The total score ranged from 24 to 120, with higher scores indicating better spiritual health. In this study, Cronbach's alpha for the scale was 0.942.

The Chinese version of the Brief Perceived Professional Benefit Questionnaire developed by Hu et al. [34] was used to measure nurses' perceived professional benefit. The scale contains 17 items in five factors: personal growth, recognition from family and friends, good patient-nurse relationship, positive professional perception, and sense of belonging to the work team. Each item was scored using a five-point Likert scale ranging from 1 (*completely disagree*) to 5 (*fully agree*). The total scores ranged from 29 to 145, with a higher score indicating greater perceived professional benefit. In this study, Cronbach's alpha for the scale was 0.979.

### 2.4. Data Analysis

The data were analyzed by IBM SPSS for Windows (version 26.0; IBM Corp, Armonk, NY, USA). Continuous data were presented as mean (standard deviation), while categorical data were expressed as frequency (percentage). Pearson's correlation coefficients were used to detect bivariate relationships among spiritual health, perceived professional benefit, and SCC. The mediation model was examined using Model four of the PROCESS macro for SPSS [49]. The model hypothesizes that *X* affects *Y* through one direct effect and one specific indirect effect. Spiritual health was set as *X*, perceived professional benefit as *M*, and SCC as *Y*. The indirect effect of *X* on *Y* via *M* is *a*_1_*b*_1_. The sum of the indirect effect (*a*_1_*b*_1_) and direct effect (*c*′) equals the total effect (*c*). Cause mediation analyses were based on the counterfactual framework, which formally defines both direct and indirect effects, and were more robust to the various limitations of traditional adjustment-based mediation analysis. Point estimates and 95% confidence intervals (CI) for indirect, direct, and total effects were calculated using bootstrapping with 5000 simulations. If the 95% CI did not contain zero, the effect was considered statistically significant.

## 3. Results

### 3.1. Sample Characteristics

A total of 336 questionnaires were distributed, of which 299 were returned, for a response rate of 89%. Of 299 nurses, the mean age was 21.05 (1.55), and 88.6% (*n* = 265) were female nurses. Most nurses were less than 24 years old (96.0%). Only 2.7% of the participants had a bachelor's degree. Most were not married (99.3%), were contract/person-employed (89.3%), had a monthly income of less than 4000 Renminbi (RMB, 95.3%), and had no religious beliefs (94.6%). Nearly one-third of the participants worked in surgical (28.1%) or internal medicine (29.1%) departments. The details are presented in Table 1.

### 3.2. Scale Scores and Correlation Coefficients

As shown in Table 2, the total scores for spiritual health, perceived professional benefit, and SCC were 95.79 (13.91), 73.18 (10.26), and 103.53 (19.57), respectively. The scores for the items of each scale were presented in File S2. Better spiritual health was correlated with perceived professional benefit (*r* = 0.655, *p* < 0.01) and SCC (*r* = 0.524, *p* < 0.01). Perceived professional benefit was positively correlated with SCC (*r* = 0.421, *p* < 0.01).

### 3.3. Mediation Effects of Perceived Professional Benefit Between Spiritual Health and SCC

**Figure 2:**
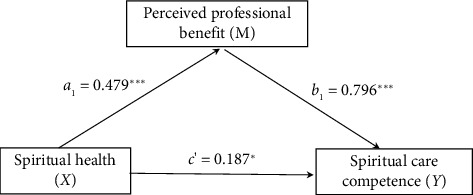
Mediation model of perceived professional benefit linking spiritual health and spiritual care competence among new nurses. ^∗^*p* < 0.05 and ^∗∗∗^*p* < 0.01.

This study assessed the mediating role of perceived professional benefit on the relationship between spiritual health and SCC. Control variables were sex, age, educational background, marital status, parental status, employment type, monthly income, religious beliefs, and department. The mediation model for spiritual health and SCC is shown in Figure 2 and Table 3. The total effect of spiritual health on SCC was significant [effect = 0.569, 95% CI (0.419–0.719)]. The direct effect of spiritual health on SCC was observed [effect = 0.187, 95% CI (0.002–0.373)]. Furthermore, spiritual health had an indirect effect on SCC via perceived professional benefit [effect = 0.382, 95% CI (0.260–0.531)], which accounted for 67.1% of the total effect. The details are presented in Table 3.

## 4. Discussion

This is the first study to report the mediating effect of perceived professional benefit on the relationship between spiritual health and SCC among new nurses. The results revealed that positive correlations were observed among spiritual health, perceived professional benefit, and SCC. Spiritual health not only directly influenced SCC but also did so indirectly through perceived professional benefit. New nurses with better spiritual health were more likely to perceive more professional benefits, which were related to higher SCC.

In this study, the new nurses experienced moderate levels of SCC, similar to the findings of Li et al. [40] and Cheng et al. [43]. During the course of this study, the threat of the COVID-19 pandemic and the implementation of special lifesaving procedures engendered a closer relationship between nurses and patients. In addition, the pandemic heightened spiritual concerns for patients, making it easier for nurses to recognize and address patients' spiritual needs, which improved nurses' SCC [40]. However, the results indicate that further improvements are required in the SCC of new nurses. Because courses in spirituality are not generally taught in medical schools and spiritual training in Chinese hospitals is inadequate [50], new nurses are ill-prepared to fulfill their responsibilities as spiritual caregivers. Therefore, it is essential to explore the associated factors of SCC and develop tailored interventions for new nurses.

Spiritual health was found to be positively associated with SCC among new nurses, which was consistent with previous studies [31, 32]. Spiritual health was an important protective factor for SCC, and a high level of spiritual health was a prerequisite for delivering high-quality spiritual care [33]. Spiritual health acts as an intrinsic motivation that facilitates nurses' provision of spiritual care [51]. Particularly in instances where patients' spiritual health conflicts with proposed medical interventions, nurses play a pivotal role in navigating these complex situations. New nurses with higher levels of spiritual health are more likely to deliver spiritual care by engaging in open communication and listening actively to understand patients' spiritual beliefs and values. Moreover, they can facilitate comprehensive discussions involving patients, their family members, healthcare providers, and spiritual counselors to explore alternatives that align with the patients' spiritual convictions while meeting their medical requirements optimally. Ultimately, patient autonomy is prioritized, advocating for a personalized care plan that respects the patient's overall well-being [52]. By using resources to address patients' spiritual needs and establishing positive connections, new nurses contribute to overall spiritual health and self-harmony [51]. Thus, interventions targeting the improvement of new nurses' spiritual health may be beneficial for promoting their SCC.

As expected, our study indicated that spiritual health influenced SCC through perceived professional benefit. New nurses with higher spiritual health perceived more professional benefits, which in turn promoted their SCC. Spiritual health is a resource that new nurses can use to cope with job-related stress for example, patients' spiritual needs. As a result, nurses are more likely to comprehend the importance of humanistic care in nursing, cultivate empathy, and enhance competency in patient care [32, 37]. By recognizing their own spiritual health, new nurses are not only better able to find meaning and purpose for themselves but also assist patients in finding meaning and purpose in their care work, thus enhancing their own spiritual care ability [36, 38]. Simultaneously, this state of spiritual health can bring positive feedback from both life and work, helping new nurses obtain a higher level of professional benefit [38, 53]. Perceived professional benefit, as a form of positive professional perceptions–for instance, a positive social image related to “saving lives” and attainment of self-worth–can help new nurses recognize the value of their nursing careers and contribute to their willingness to make sustained efforts, ultimately improving their SCC [36, 40]. Therefore, it can be said that the spiritual health of new nurses indirectly enhances their spiritual care ability through their perception of professional benefit. Despite the lack of conclusive evidence for a mediating effect, our findings are supported by prior research, suggesting that perceived professional benefit at least partially explains the relationship between spiritual health and SCC among new nurses. Our findings highlight the importance of perceived professional benefit, particularly for new nurses who have a low level of spiritual health. Hospital administrators should prioritize nurses' perceived professional benefit from a positive psychological perspective, undertake interventions that evaluate occupational cognition, and assist nurses in realizing their worth in improving new nurses' SCC.

### 4.1. Strengths and Limitations

Our study has several strengths. First, it reports on SCC among new nurses, which studies have rarely addressed. Second, the C-SCCS used in this study demonstrates good reliability and validity. Third, our study tests the mediating effect of perceived professional benefit on the relationship between spiritual health and SCC among new nurses. These findings contribute to our understanding of SCC among new nurses who have recently entered clinical nursing practice. Cultivating SCC among new nurses is critical for providing holistic care and optimizing nursing quality. Therefore, our findings provide an empirical basis for future intervention strategies aimed at improving SCC among new nurses.

Some limitations of this study need to be acknowledged. First, due to the study's cross-sectional design, causation could not be established. Therefore, longitudinal or interventional studies are warranted. Second, the participants were recruited from Henan Province using convenience sampling, which limited the generalizability of the present findings. Future studies should use random samples from different hospitals and regions. Third, self-report questionnaires were used, and response bias by the participants may have occurred.

## 5. Conclusion

The level of SCC of new nurses was moderate. Perceived professional benefit mediated the relationship between spiritual health and SCC among new nurses. Thus, it is necessary for healthcare managers to implement interventions that focus on promoting spiritual health and then increasing perceived professional benefit to improve SCC among new nurses.

## 6. Relevance to Clinical Practice

This study measured the SCC of new nurses and tested the mediating role of perceived professional benefit in the relationship between spiritual health and SCC. The findings indicate the importance of promoting new nurses' SCC in China. Healthcare managers can promote their SCC both directly by cultivating their spiritual health and indirectly by enhancing their professional benefit. New nurses' spiritual health can be improved through spiritual care education and training programs such as lecture-based learning, group discussion, and simulated clinical skill practice [18, 54]. Furthermore, interventions of occupational cognitive evaluation are advantageous for improving nurses' perceptions of their professional benefits [55]. Improvements in SCC among new nurses are beneficial for promoting patients' spiritual health and optimizing nursing quality.

## Figures and Tables

**Table 1:** Characteristics of samples (*n* = 299).

Variable	Mean (SD)/*n* (%)
Age (years)	21.05 (1.55)

*Gender*	
Male	34 (11.4)
Female	265 (88.6)

*Educational background*	
Junior college degree	291 (97.3)
Bachelor's degree	8 (2.7)

*Marital status*	
Unmarried	297 (99.3)
Married	2 (0.7)

*Parental status*	
No	292 (97.7)
Yes	7 (2.3)

*Employment type*	
Contract-employed	233 (77.9)
Personnel agency-employed	34 (11.4)
State-employed	32 (10.7)

*Monthly income (RMB)*	
< 4000	285 (95.3)
≥ 4000	14 (4.7)

*Religious beliefs*	
No	283 (94.6)
Yes	16 (5.4)

*Department*	
Internal medicine	87 (29.1)
Surgery	84 (28.1)
Obstetrics and gynecology	40 (13.4)
Pediatrics	24 (8)
Outpatient and emergency	23 (7.7)
Others	41 (13.7)

Abbreviation: RMB, renminbi.

**Table 2:** Mean scores and correlation coefficients of study variables.

Variable	Mean (SD)	1	2	3
Spiritual health	95.79 (13.91)	1		
Perceived professional benefit	73.18 (10.26)	0.655^∗∗^	1	
Spiritual care competence	103.53 (19.57)	0.524^∗∗^	0.421^∗∗^	1

Abbreviation: SD, standard deviation.

^∗∗^ *p* < 0.01.

**Table 3:** Mediation analysis of spiritual health and spiritual care competence.

	Effect	SE	*p*	LLCI	ULCI
Total effect	0.569	0.076	< 0.001	0.419	0.719

*Direct effect*					
Spiritual health ⟶ spiritual care competence	0.187	0.094	0.047	0.002	0.373

*Indirect effect*					
Spiritual health ⟶ perceived professional benefit ⟶ spiritual care competence	0.382	0.070	—	0.260	0.531

*Note:* Adjusting for covariates, including age, sex, educational background, marital status, parental status, employment type, monthly income, religious beliefs, and department.

Abbreviations: LLCI, lower limit confidence interval; SE, standard error; ULCI, upper limit confidence interval.

## Data Availability

The data that support the findings of this study are available from the corresponding author upon reasonable request.

## Supporting Information

Supporting Information 1 Supporting File S1: STROBE Statement—Checklist of items that should be included in reports of cross-sectional studies.

Supporting Information 2 Supporting File S2: Table S1 Descriptive analyses of study variables (*n* = 299).
