# Supplementary material for: Mediating Effect of Perceived Professional Benefit on the Relationship Between Spiritual Health and Spiritual Care Competence Among New Nurses: A Cross-Sectional Study
Source: J Nurs Manag. 2025 May 2;2025:8832454. doi: 10.1155/jonm/8832454 (PMC12064319; doi:10.1155/jonm/8832454)
Supplement: Supporting Information 2 — Supporting File S2: Table S1 Descriptive analyses of study variables (n = 299). [file 8832454.f2.docx]

Table S1: Descriptive analyses of study variables (n=299).

| Items | Min. | Max. | M | SD |
| --- | --- | --- | --- | --- |
| **Spiritual health** | 24 | 120 | 95.79 | 13.91 |
| ***Connections to others*** | 4 | 20 | 17.63 | 2.88 |
| Item 1 I can have intimate talks with family members | 1 | 5 | 4.26 | 1.00 |
| Item 2 I like to help solve problems for family members | 1 | 5 | 4.39 | 0.78 |
| Item 3 When I am in a difficult situation, I can often get help from my family members | 1 | 5 | 4.49 | 0.74 |
| Item 4 I feel that a harmonious relationship with family is very important | 1 | 5 | 4.49 | 0.75 |
| ***Meaning derived from living*** | 6 | 30 | 25.98 | 4.06 |
| Item 5 I think about how to have a more fulfilled life | 1 | 5 | 4.32 | 0.73 |
| Item 6 I think about what my long-term goals in life are | 1 | 5 | 4.32 | 0.75 |
| Item 7 I try my best to live a meaningful life | 1 | 5 | 4.39 | 0.73 |
| Item 8 I engage in career planning | 1 | 5 | 4.26 | 0.78 |
| Item 9 I try my best to play my role in life | 1 | 5 | 4.30 | 0.79 |
| Item 10 I devote effort to completing my long-term goals in life | 1 | 5 | 4.38 | 0.72 |
| ***Transcendence*** | 6 | 30 | 26.00 | 4.23 |
| Item 11 I see setbacks as a type of challenge | 1 | 5 | 4.35 | 0.73 |
| Item 12 When I encounter setbacks, I can boldly face them without fear | 1 | 5 | 4.26 | 0.78 |
| Item 13 When I encounter setbacks, I believe I have the ability to self-recover | 1 | 5 | 4.29 | 0.80 |
| Item 14 I face setbacks in life with a proactive and optimistic attitude | 1 | 5 | 4.36 | 0.76 |
| Item 15 Even if I suffer a setback, I still believe life is beautiful | 1 | 5 | 4.36 | 0.77 |
| Item 16 I can experience my own abilities from setbacks | 1 | 5 | 4.38 | 0.77 |
| ***Religious attachment*** | 4 | 20 | 9.15 | 4.92 |
| Item 17 I hope to be blessed by God (i.e., God, heavenly god, deity, Buddha, or Bodhisattva) | 1 | 5 | 2.64 | 1.36 |
| Item 18 I believe religion or religious beliefs can help me overcome obstacles | 1 | 5 | 2.19 | 1.29 |
| Item 19 Religious activities (such as praying, asking deity for understanding, worshipping, etc.) are very helpful for me | 1 | 5 | 2.11 | 1.28 |
| Item 20 I believe in the blessing from God (i.e., God, heavenly god, deity, Buddha, or Bodhisattva), and it gives me peace | 1 | 5 | 2.21 | 1.30 |
| ***Self-understanding*** | 4 | 20 | 17.03 | 2.72 |
| Item 21 I can appreciate my own merits | 1 | 5 | 4.19 | 0.76 |
| Item 22 I can accept my shortcomings | 1 | 5 | 4.23 | 0.76 |
| Item 23 I like myself | 1 | 5 | 4.30 | 0.78 |
| Item 24 I believe I am a person with value | 1 | 5 | 4.31 | 0.75 |
| **Perceived professional benefit** | 17 | 85 | 73.18 | 10.26 |
| ***Positive professional perception*** | 3 | 15 | 12.53 | 2.25 |
| Item 1 I think it is good to be a nurse | 1 | 5 | 4.09 | 0.82 |
| Item 2 The nursing profession allows me to develop my strengths and realize my social value | 1 | 5 | 4.19 | 0.78 |
| Item 3 I am proud of the good image of the nursing profession as being people’s ‘white angel’ and ‘healing the wounded | 1 | 5 | 4.24 | 0.78 |
| ***Good patient-nurse relationship*** | 4 | 20 | 17.50 | 2.58 |
| Item 4 The praise or gratitude of the patient and his/her family after successfully saving the patient’s life has improved my sense of professional value | 1 | 5 | 4.33 | 0.68 |
| Item 5 The patient improved/healed under my care, giving me a sense of accomplishment | 1 | 5 | 4.38 | 0.67 |
| Item 6 I am very happy to be able to help patients at work | 1 | 5 | 4.39 | 0.67 |
| Item 7 I am pleased that patients can understand my work | 1 | 5 | 4.41 | 0.67 |
| ***Recognition from family members, relatives, and friends*** | 3 | 15 | 12.81 | 1.93 |
| Item 8 The nursing profession allows me to provide convenient medical resources for my relatives and friends | 1 | 5 | 4.16 | 0.81 |
| Item 9 I can provide professional care when my family is sick | 1 | 5 | 4.33 | 0.68 |
| Item 10 When the family is sick, I can provide them with professional guidance (including medical treatment, medication, etc. | 1 | 5 | 4.31 | 0.70 |
| ***Sense of belonging to a team*** | 3 | 15 | 12.92 | 1.94 |
| Item 11 I can get guidance and affirmation from the leadership at work. | 1 | 5 | 4.26 | 0.70 |
| Item 12 Mutual communication with colleagues enhances my confidence and strength. | 1 | 5 | 4.32 | 0.67 |
| Item 13 My work team can help each other and gets along well, making me feel warm | 1 | 5 | 4.34 | 0.66 |
| ***Self-growth*** | 4 | 20 | 17.43 | 2.45 |
| Item 14 I often encounter unexpected situations in my work, which improves my psychological quality (such as coordination, resilience, etc | 1 | 5 | 4.36 | 0.63 |
| Item 15 The nursing profession has taught me how to deal with various people and enhance my interpersonal skills. | 1 | 5 | 4.34 | 0.63 |
| Item 16 As my professional skills continue to improve, my professional mentality is maturing | 1 | 5 | 4.36 | 0.62 |
| Item 17 The nursing profession has made me develop a patient and meticulous style | 1 | 5 | 4.36 | 0.62 |
| **Spiritual care competence** | 27 | 135 | 103.53 | 19.57 |
| ***Assessment, implementation, professionalization and quality improvement of spiritual care*** | 12 | 60 | 46.69 | 8.99 |
| Item 1 I can report orally and/or in writing on a patient's spiritual needs | 1 | 5 | 3.79 | 0.86 |
| Item 2 I can tailor care to a patient's spiritual needs/problems in consultation with the patient | 1 | 5 | 3.95 | 0.78 |
| Item 3 I can tailor care to a patient's spiritual needs/problems through multidisciplinary consultation | 1 | 5 | 3.95 | 0.79 |
| Item 4 I can record the nursing component of a patient's spiritual care in the nursing plan | 1 | 5 | 3.97 | 0.81 |
| Item 5 I can report in writing on a patient's spiritual functioning | 1 | 5 | 3.9 | 0.82 |
| Item 6 I can report orally on a patient's spiritual functioning | 1 | 5 | 3.87 | 0.83 |
| Item 7 Within the nursing ward, I can contribute to quality assurance in the area of spiritual care | 1 | 5 | 3.87 | 0.86 |
| Item 8 Within the nursing ward, I can contribute to professional development in the area of spiritual care | 1 | 5 | 3.90 | 0.81 |
| Item 9 Within the nursing ward, I can identify problems relating to spiritual care in peer discussion sessions | 1 | 5 | 3.87 | 0.86 |
| Item 10 I can coach other care workers in the area of spiritual care delivery to patients | 1 | 5 | 3.88 | 0.85 |
| Item 11 I can make policy recommendations on aspects of spiritual care to the management of the nursing ward | 1 | 5 | 3.87 | 0.85 |
| Item 12 I can implement a spiritual care improvement project in the nursing ward | 1 | 5 | 3.87 | 0.86 |
| ***Personal and team support*** | 9 | 45 | 33.29 | 7.74 |
| Item 13 I can provide a patient with spiritual care | 1 | 5 | 3.82 | 0.87 |
| Item 14 I can evaluate the spiritual care that have provided in consultation with the patient and with the disciplinary/ multidisciplinary team | 1 | 5 | 3.81 | 0.87 |
| Item 15 I can give a patient information about spiritual facilities within the care institution (including spiritual care meditation centres, religious services | 1 | 5 | 3.64 | 1.00 |
| Item 16 1 can help a patient continue his or her daily spiritual practices including providing opportunities for rituals. prayer, meditation, reading the Bible/Koran, listening to music | 1 | 5 | 3.6 | 1.02 |
| Item 17 I can attend to a patient's spirituality during daily care (e.g., physical care) | 1 | 5 | 3.76 | 0.91 |
| Item 18 I can refer members of a patient's family to a spiritual adviser/pastor, etc, if they ask me and/or if they express spiritual needs | 1 | 5 | 3.6 | 1.03 |
| Item 19 I can effectively assign care for a patient's spiritual needs to another care provider/care worker/care discipline | 1 | 5 | 3.73 | 0.93 |
| Item 20 At the request of a patient with spiritual needs, I can in a timely and effective manner refer him or her to another care worker (e.g., a chaplain/the patient's own priest/imam) | 1 | 5 | 3.65 | 1.00 |
| Item 21 I know when I should consult a spiritual adviser concerning a patient's spiritual care | 1 | 5 | 3.69 | 0.89 |
| ***Attitude towards patient spirituality and communication*** | 6 | 30 | 23.55 | 4.71 |
| Item 22 I show unprejudiced respect for a patient's spiritual/religious beliefs regardless of his or her spiritual/religious background | 1 | 5 | 3.92 | 0.84 |
| Item 23 I am open to a patient's spiritual/religious beliefs, even if they differ from my own | 1 | 5 | 3.93 | 0.82 |
| Item 24 I do not try to impose my own spiritual/religious beliefs on a patient | 1 | 5 | 3.97 | 0.88 |
| Item 25 I am aware of my personal limitations when dealing with a patient’s spiritual/religious beliefs | 1 | 5 | 3.79 | 0.90 |
| Item 26 I can listen actively to a patient's 'life story' in relation to his or her illness/handicap | 1 | 5 | 3.96 | 0.83 |
| Item 27 I have an accepting attitude in my dealings with a patient concerned, sympathetic,inspiring trust and confidence, empathetic, genuine sensitive. sincere and personal | 1 | 5 | 3.97 | 0.84 |

Note: M：mean; SD：Standard deviation
